# Sampling and detection of airborne influenza virus towards point-of-care applications

**DOI:** 10.1371/journal.pone.0174314

**Published:** 2017-03-28

**Authors:** Laila Ladhani, Gaspard Pardon, Hanne Meeuws, Liesbeth van Wesenbeeck, Kristiane Schmidt, Lieven Stuyver, Wouter van der Wijngaart

**Affiliations:** 1 KTH Royal Institute of Technology, Department of Micro and Nanosystems, Stockholm, Sweden; 2 Janssen Diagnostics, Beerse, Belgium; University of Alabama at Birmingham, UNITED STATES

## Abstract

Airborne transmission of the influenza virus contributes significantly to the spread of this infectious pathogen, particularly over large distances when carried by aerosol droplets with long survival times. Efficient sampling of virus-loaded aerosol in combination with a low limit of detection of the collected virus could enable rapid and early detection of airborne influenza virus at the point-of-care setting. Here, we demonstrate a successful sampling and detection of airborne influenza virus using a system specifically developed for such applications. Our system consists of a custom-made electrostatic precipitation (ESP)-based bioaerosol sampler that is coupled with downstream quantitative polymerase chain reaction (qPCR) analysis. Aerosolized viruses are sampled directly into a miniaturized collector with liquid volume of 150 μL, which constitutes a simple and direct interface with subsequent biological assays. This approach reduces sample dilution by at least one order of magnitude when compared to other liquid-based aerosol bio-samplers. Performance of our ESP-based sampler was evaluated using influenza virus-loaded sub-micron aerosols generated from both cultured and clinical samples. Despite the miniaturized collection volume, we demonstrate a collection efficiency of at least 10% and sensitive detection of a minimum of 3721 RNA copies. Furthermore, we show that an improved extraction protocol can allow viral recovery of down to 303 RNA copies and a maximum sampler collection efficiency of 47%. A device with such a performance would reduce sampling times dramatically, from a few hours with current sampling methods down to a couple of minutes with our ESP-based bioaerosol sampler.

## Introduction

Presently, influenza remains a serious threat to public health. It is a rapidly spreading and highly contagious respiratory infection that results not only in yearly epidemics but also in intermittent pandemics. Many of the influenza transmission events are suspected to occur via aerosolized virus [1][2], traveling via self-contained liquid droplets of < 5 μm diameter [3–5]. These small droplets can remain airborne for long periods, ranging from minutes to hours, resulting in far-reaching spread of airborne infection. Additionally, sub-micrometer aerosol droplets are within the size range of particles that are likely to be deposited in the lower airways of a subject during inhalation, increasing the risk of infection [6]. Common measures against infection, such as improved hand hygiene or facial masks, are inefficient against transmission via such small droplets [7]. A recent report by the Institute of Medicine [8] expresses the urgent need for expedited research on influenza transmission to develop “effective prevention and control strategies” during an influenza epidemic.

Our work addresses this need and proposes a technology for the detection of airborne pathogens for air monitoring and breath-based diagnostics. More specifically, we aim to improve schemes and devices that enable detection at the point-of-care (PoC) setting. Such technologies should enable optimal application in automated PoC devices and these devices should be small and easy to use. In addition, they should easily interface the air sample with the lab-on-chip (LoC) biosensors. With respect to the latter concern, it is preferable that viruses be sampled directly into a liquid solution. There are three main reasons for this: 1) most established bioanalytical techniques are liquid-based detection schemes (cell culture, antibody-based detection, nucleic acid detection, etc.); 2) sampling into liquid has been shown to preserve viral integrity better than collecting onto dry solid surfaces; 3) and collection into liquid allows interfacing with a disposable LoC containing a suitable bioassay and biosensor, enabling the detection of viral particles at the PoC setting. Furthermore, increased sensitivity is needed to detect the low viral load present in aerosols. Therefore, a low limit of detection (LoD) of the PoC scheme must be addressed. Low LoDs can be achieved by considering one or a combination of the following parameters: increased sensitivity of the bioassay, increased sampling efficiency of the device, increased sample recovery from the device, increased sampling time, and/or a decreased collection volume. The latter minimizes the dilution of virus into unnecessarily large liquid volumes, yielding high a concentration of recovered virus, and therefore contributing to lowering the system LoD for a given biosensor LoD. Indeed, a sampling device displaying high collection efficiency may seem impressive, but it will not prove more useful in terms of sampling duration or lowering the limit of detection than a less efficient device if the internally engendered sample dilution is comparatively much larger. Based on the previous considerations, we aim for a device design that not only works well with a standard well-established bioassay but also minimizes dilution and internal losses of the sampled viruses while maintaining a sufficiently high physical sampling efficiency.

The three most common bioaerosol sampler types are impingers, filters, and impactors. Impingers are liquid collection-based samplers relying on working volumes of 5 or 20 mL, whereas filters and impactors are dry collection-based samplers relying on an elution step to extract the collected virus from their respective solid supports. This elution can result in a large final extracted sample volume, typically on the order of several milliliters [6, 9–11]. These large volumes engender enormous dilution if only a few viral particles are present in the sampled volume of air, making these methods unsuitable for low LoD measurements. Moreover, integrating and automating the handling and processing of a liquid volume from either an impinger, filter, or impactor to a biosensor requires substantial system complexity unsuited for PoC applications.

Alternatively, electrostatic precipitators (ESPs) have been reported recently as bioaerosol samplers for the collection of airborne microorganisms [3, 12–15]. ESPs benefit from low power consumption and a gentler sampling that is potentially less damaging for pathogens [14]. Recently, Pardon et al. [16] proposed an ESP device design for sampling airborne particles directly onto a microfluidic air-liquid interface. This approach has the potential for adaptation to PoC settings. However, sampling and recovery efficiencies for such an approach have not yet been tested for aerosolized virus particles. Here, we demonstrate, for the first time, efficient sampling of aerosolized influenza virus directly into a miniaturized liquid volume and low-limit detection for sub-micrometer-sized aerosol. This sampling is achieved using our custom-made ESP sampler featuring an improved functional design, compared to previously reported designs [16], in conjunction with a downstream quantitative polymerase chain reaction (qPCR) assay.

## Experimental approach

The main purpose of our experiments is to demonstrate a technical solution suitable for the efficient sampling of airborne influenza virus and compatible with sensitive detection at the point-of-care setting. To that end, we propose a custom-designed ESP-based sampler combined with downstream qPCR analysis for sensitive evaluation. We assess the LoD and collection efficiency of the combined sampling-and-sensing scheme, *η*_*coll*_.

To evaluate our approach, we use an aerosol that represents the characteristics of real-world airborne influenza virus, i.e. sub-micrometer virus-loaded aerosol droplets, nebulized with a size range chosen to be as representative as possible of the aerosol characteristics reported for exhaled breath (see Methods and Supplementary information, S2 Fig) [17–19].

The ESP-based sampling device, whose design details are described below, was optimized with respect to the two following parameters:

- Physical sampling efficiency, *η*_*samp*_, which is the ratio of viruses sampled over the total number of airborne viruses available in the air stream.
- Viral extraction efficiency, *η*_*extr*_, which is the ratio of viruses effectively reaching the biosensor over the total number of sampled viruses.

**Fig 1:**
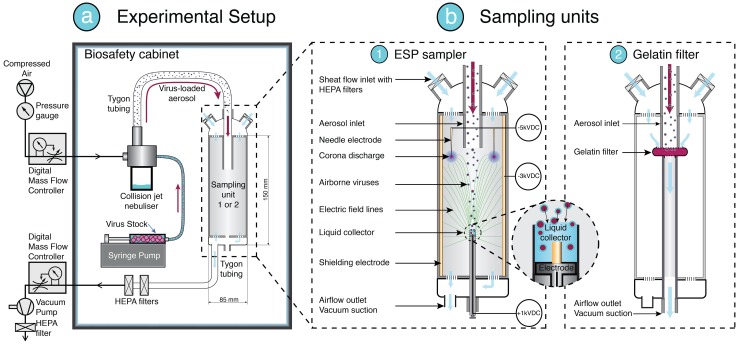
Schematics of the experimental setup (not to scale). (a) Experiments are conducted in a bio-safety cabinet to keep all aerosolized virus contained. The setup includes a Collison nebulizer to aerosolize using compressed air and viral solutions provided by a syringe pump. Tubing directs the airborne viruses into the sampling unit (b): (1) our ESP sampler or (2) gelatin filters for comparison. A downstream vacuum pump maintains a constant flow and under pressure inside the ESP sampler. (b) Details of the sampling units: (1) Our ESP sampler includes a three-electrode corona discharge electrostatic precipitator to capture the aerosol particle directly into an integrated liquid collector with a miniaturized volume of 150 μL; (2) gelatin filters are used for comparative measurement of the total amount of virus effectively entering the ESP sampler after nebulization. The filters are housed in a specific cassette placed at the aerosol inlet.

The physical sampling efficiency and viral extraction efficiency are difficult to assess independently from one another. However, the system collection efficiency, *η*_*coll*_ = *η*_*samp*_ *x η*_*extr*_, was determined experimentally. Further details of the ESP sampler principle and design as well as investigation of the two efficiencies are described below. Schematics of the setup and the sampler are shown in Fig 1.

### Principle and design of ESP sampler

Electrostatic precipitation relies on the following principle: a potential difference is applied between two electrodes, one with high curvature and one with low curvature, creating an electric field in the inter-electrode space, which precipitates any charged particles by exerting an electric force on them. Additionally, applying a high voltage to the high curvature electrode produces a corona discharge; in our sampler, these electrodes are multiple sharp needles with tips that produce a local corona discharge. This corona discharge ionizes the air molecules surrounding the needle tips and any incoming aerosol droplets are electrically charged upon colliding with these ions. The charged aerosol droplets are then electrophoretically transported towards the low curvature counter electrode; in our sampler, this is the liquid collector. The design (Fig 1_b_1) of our ESP sampler is an improved version of the device design proposed by Pardon et al. [16] (further details provided in Methods and in the referenced article). Our design is characterized by three main improvements: 1) a two times reduction of the collection volume; 2) the addition of a third electrode at the inner wall of the device housing to allow better focusing of the electric field to the liquid collector, improving the capture of incoming particles compared to the previously reported design for enhanced control of the electrostatic precipitation; 3) and a reduction of the overall dimensions of the sampler, making it compact and suitable for point-of-care use.

### Physical sampling efficiency

To determine the overall sampling efficiency of the ESP sampler, the amount of aerosolized virus entering the device needs to be known. Currently, no gold standard method exists for generating bioaerosols with known viral load; instead, we generate aerosols from liquid with different viral concentration and measure the resulting viral load of these aerosols at the device entrance. Accordingly, the system collection efficiency, *η*_*coll*_, is determined as the ratio between the amount of virus captured with our ESP sampler, #Virus_ESP_, and the amount of virus entering the sampler, #Virus_filter_, under equal operating conditions:

$$\eta_{coll}=\;\eta_{samp}\cdot \eta_{extr}=\;\frac{{\text{\#Virus}}_{\text{ESP}}}{{\text{\#Virus}}_{\text{filter}}}.$$ (1)

To verify the effect of active sampling due to electrostatic affects, control experiments were performed for several viral stock dilutions, while the ESP sampler voltage supplies were turned off, i.e. in absence of corona discharge and electrostatic precipitation.

### Viral extraction efficiency

We hypothesized that precipitated virus could remain adsorbed onto the collector surfaces, potentially leading to reduced viral recovery. Therefore, we investigated three extraction protocols (EPs) for removing a sample from the liquid collector to evaluate the losses during the extraction step.

EP1: Liquid pipetting
  Liquid sample was extracted using manual pipetting.
EP2: Liquid pipetting + UTM rinsing
  As in EP1, the liquid sample was extracted using manual pipetting. Next, a 225 μL of universal transport medium (UTM) was used to rinse all inner and outer surfaces of the collector using a pipette.
EP3: Liquid pipetting + Filter wiping + UTM rinsing
  As in EP1, the liquid sample was extracted using manual pipetting. Any remaining liquid was soaked up with a gelatin filter, and a second filter was used to wipe the outer surface of the collector. Finally, the collector was rinsed with 150 μL of UTM.

Sample extraction was also analyzed for gelatin filters, showing close to 100% extraction efficiency (see Supplementary information).

## Materials and methods

**Fig 2:**
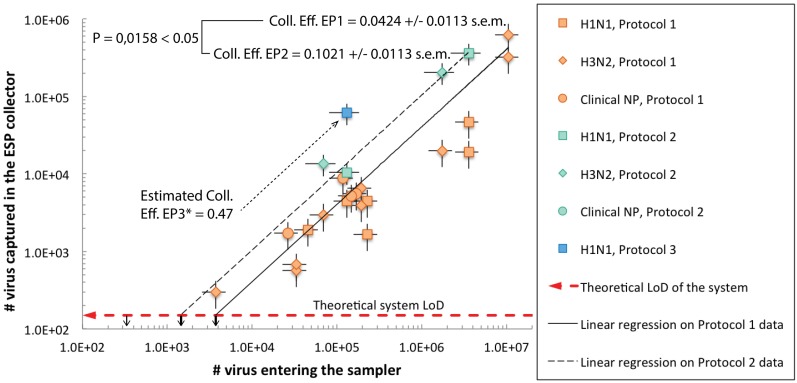
Measurements of the total amount of viruses detected with qPCR versus the total amount of viruses entering the ESP sampler. Linear regressions were performed using the least-square method on data groups obtained using EP1 and EP2. The qPCR LoD reported to the sampling volume is plotted with a horizontal dashed red arrow and enables calculating the theoretical system LoDs, indicated with black arrows. The collection efficiencies for EP1 and EP2 are significantly different with a probability P = 0.0158 < 0.05 and are indicated with the corresponding standard error. *For EP3, only a single measurement was successfully obtained. The corresponding collection efficiency can be estimated using Eq 1.

Our experimental protocol consisted of a concatenation of four steps: 1) aerosolization of influenza virus using a Collison nebulizer; 2) sampling directly into a liquid collector (volume *V* = 150 μL) using our ESP sampler (Fig 2_b_1) or onto a gelatin filter (Fig 2_b_2); 3) extraction of the collection liquid using various extraction protocols (EP); and 4) analysis of collected samples using qPCR.

### Influenza virus aerosol

Three types of virus stock were used: H3N2 subtype of clinical nasopharyngeal (NP) swab samples and two subtypes of cultured virus–H1N1(A/Puerto Rico/8/1934) and H3N2 (A/Aichi/2/1968). For the NP swab samples, four patient mid-turbinate flocked swab samples (H3N2) from the Belgium Prospective study (2011–2012) were used. (The study was approved by the Committee on Medical Ethics of the University Hospital of KULeuven; written consent was obtained from all participants [21]). These stocks were used in a 1 to 10 dilution in UTM. From the latter two, ten-fold serial dilutions were prepared using UTM transport buffer (COPAN), i.e. ranging from 3 log_10_ copies/mL to 9 log_10_ copies/mL. Aliquots were stored at -80°C until further testing.

Measurements for each serial dilution of the cultured samples were performed in duplicate, although the clinical NP swab samples were measured only once due to scarcity. An experiment (nebulization, sampling, and extraction) was done for each dilution series sequentially, starting from the sample with lowest viral load. Aerosol droplets were generated using the BANG (Bio-Aerosol Nebulizing Generator) nebulizer (CH Technologies, USA), based on the Collison nebulizing principle. The nebulizer was operated in single-pass mode to generate an aerosol with a log-normal size distribution with a geometric diameter of 0.371 μm and a standard deviation of 1.371, leading to a mean mass diameter (MMD) of 1.074 μm and a diameter of average mass of 0.482 μm. (see Supplementary information, S1 Fig). A GRIMM optical particle counter (OPC) (GRIMM Aerosol Technik GmbH & Co., DE) was used to determine droplet sizes of the generated aerosols.

### Laboratory setup

Nebulization and sampling was performed in a bio-safety cabinet (Fig 2). All instrument parts potentially in contact with viruses were carefully kept inside the bio-safety cabinet, and all extraneous instruments were placed outside. A mass flow controller (MFC) (Bronkhorst High-Tech B.V., NL) was used to control flow rate of the compressed air supplied to the nebulizer, while a syringe pump (model 540060, TSE Systems GmbH, DE) was used to set the liquid sample feed rate to the nebulizer. Tygon tubing (Sigma-Aldrich, USA) was used to deliver aerosol samples from the nebulizer to the ESP sampler and to connect the sampler outlet to a vacuum pump whose flow was controlled using a second MFC. HEPA filters (Filta-Therm HMEF, Intersurgical Ltd., UK) were installed in line downstream from the ESP sampler to prevent any uncaptured viral aerosol from exiting the safety cabinet, ensuring a completely contained and leak-free environment.

### ESP sampler

The basic feature of our ESP sampler was built following design guidelines previously published [16]. The following improvements were made to this original design:

- A Stratasys μPrint SE printer (Statrasys Ltd., UK) was used to 3D-print the body, inlet, and outlet (Fig 1_b_1).
- In addition to the previously used needle-collector two-electrode configuration, a third electrode was added to the inner wall of the ESP body to improve the electrostatic field distribution in the sampler. For this, a stainless steel metal foil (Georg Martin GmbH, Germany) was wrapped and electrically connected.
- The needle-collector inter-electrode distance (ID) was fixed to 3 cm after optimization and in accordance to Pardon et al.
- The operational voltages for the corona discharge, collector, and wall electrodes were fixed at -5.5 kV, +2 kV, and -2.7 kV, respectively. The wall electrode helps focus the electric field lines towards the collector electrode, which improves the sampling efficiency. Currents and voltages were continuously monitored and showed consistent values throughout the experiments.
- The collector electrode, a liquid container assembly, was produced as a disposable unit to alleviate the risk of cross-contamination between experiments. This unit was built using a modified low-density polyethylene (LDPE)–polystyrene (PS) dose syringe (Qosina, USA) of which the top-cylinder perforated-cap was removed and to which a gold-plated nickel electrode and connection wire were added. The syringe piston enables setting the collector volume to the desired amount, i.e. 150 μL, and to alleviate spilling of the liquid sample during the insertion and extraction of the unit from/in the ESP. The syringe collector had a 10-mm outer diameter and 8.6-mm inner diameter.
- The overall device dimensions of the cylindrical ESP sampler were decreased, with a 140-mm diameter and a 150-mm height.
- HEPA filters (Camfil Svenska AB, SE) were custom-cut using a laser plotter and were incorporated at the sheath flow inlet and at the outlet of the ESP sampler body to ensure laminar flow.

### Determining the viral load of the aerosol entering the device

To make the assessment of the collection efficiency independent from the nebulization apparatus and from the aerosol transport between the nebulizer and the ESP inlet, we generated virus-loaded aerosols in a range of stock solutions with different viral loads. For each stock viral load aerosolized, we quantified the amount of virus at the entrance of the ESP sampler by placing gelatin filters at the inlet (Fig 1_b_2). Gelatin filters were chosen as a reference sampling method because of their reported collection efficiency (>99.9%) for airborne viruses [20]. Note that despite their high collection efficiency, gelatin filters are not ideal for sampling aerosolized virus due to the large volume extraction method and extremely dry sampling environment within the filter, which could affect the integrity of the virus particle.

To determine the total viral input at the aerosol inlet of the ESP sampler, the ESP was adapted to house disposable filter cassettes. The cassettes (Sartorius Ltd., UK) were used to house 25-mm gelatin filters (ProCare BV, The Netherlands), and their outlet was directly connected to the vacuum pump. To ensure optimal sampling, a downstream flow rate was maintained at 3 L/min using an MFC.

### ESP-sampling procedure

A syringe collector was loaded with 150 μL of UTM and inserted into the ESP sampler. The vacuum pump was turned on and the downstream MFC was set to maintain a downstream flow at 6.8 L/min, effectively simultaneously controlling the sheath flow rate. The voltage modules were turned on, and corona currents were fixed at 0.6 μA throughout the sampling time. The upstream MFC was used to monitor flow rate of the pressurized air input to the nebulizer, and kept at 1 bar (Aerosol A) or 2 bar (Aerosol B). A 1-mL syringe was loaded with influenza virus, and using a syringe pump, 700 μL were injected into the nebulizer at a rate of 50 μL/min, resulting in a total aerosolization time of 14 minutes. After the syringe pump, and subsequently the pressurized air, was switched off to stop the aerosolization process, the electrical connections were switched off to stop the electrostatic precipitation. The vacuum pump was left running for one extra minute to ensure that any remaining airborne particles were sucked out of the ESP sampler into the output HEPA filters. The total experiment time was 15 minutes. The syringe collector was then removed from the device and the liquid sample was extracted using Extraction Protocols 1, 2, or 3. Of the 150 μL of UTM initially loaded into the collector, 120 μL remained as 30 μL was lost due to evaporation. This minimal evaporation in our device is due to the small air-liquid surface area exposed.

### Filter-sampling procedure

To determine the total viral input, the same protocol was used, except for the ESP sampler being replaced by the gelatin filters. The filters were extracted and kept dry in a 10-mL tube at -80 C until analysis.

### Cross-contamination control

To eliminate cross-contamination between samples of different viral loads or viral subtypes, experiments with aerosol generated from pure UTM were interposed–we refer to these as *blank control samples*. UTM (6 mL) was nebulized into the setup, both in the gelatin filters and in the ESP sampler, which were stored and analyzed in the same way as the influenza virus samples.

### Sample analysis

All viral stock dilutions prepared for aerosolization and all samples extracted from the liquid collector and gelatin filter went through an RNA extraction step. Next, a RT-qPCR analysis was performed. Nucleic acids were isolated starting from 100 μl (out of 120 μl) for the ESP samples or 1000 μl (out of 1500 μl) for the filter samples using the EasyMAG extraction platform (BioMérieux). An Internal Extraction Control (IEC) was added to all samples before the RNA extraction, as described previously [21,22]. The RNA was always eluted in 100 μl and stored at -80°C until further processing. RT-qPCR (5 μl RNA input) was performed according to the CDC protocol for influenza A virus (targeting the Matrix gene) with a panel of oligonucleotide primers and dually labelled hydrolysis (TaqMan^®^) probes, as previously described [21,23], with the change of the 25 μl IEC reaction mixture of the qPCR comprised of 5 μl of nucleic acid, 12.5 μl of 2x PCR master mix, 0.5 μl of Platinum^®^ Taq Mix (Life Technologies), 0.5 μl of forward and reverse primers (40 μM), 0.5 μl of labelled probe (10 μM), and 5.5 μl of water.

Amplification and detection were performed on a Lightcycler^®^ 480 instrument (Roche Applied Science). Each sample was tested in duplicate. All Cq values were corrected for the loss of RNA during RNA extraction using the IEC [22]. In this study, all Cq values were corrected using the average IEC of all experiments with exception of all filter sample values. A standard RNA dilution series (External quantification control; EQC) was tested in duplicate in each RT-qPCR experiment [21].

The viral loads of the processed samples were calculated as the log_10_ copies/ml using the EQC standard curve, which has a linear range from 4.3 to 10.3 log_10_ copies/ml (lower and upper limit of quantification (LLOQ and ULOQ)). Samples with an influenza viral load below LLOQ, but with a detectable amount of influenza virus RNA, were extrapolated. Corrections for RNA extraction input volume were performed. Next, correction for initial volume was performed to determine the values of absolute RNA log_10_ copies detected.

### Data analysis

Data were grouped according to the EP used in the experiments, leading to two data groups of sizes n_i_ = 19 and n_i_ = 4. EP3 provided only one useful measurement, so no statistical verification could be performed and its value is merely indicative.

An analysis of covariance (one-way ANOCOVA) was performed on the first two groups, i.e. data from EP1 and EP2, using the aoctool statistical function of the MATLAB software, fitting separate lines using the least-squared method on grouped data and returning the fitting parameters as well as the results of the analysis of covariance (See Supplementary Information S3 Table and S4 Fig). A α-level of 0.05 was used in both the t-test of the regression coefficients and in the F-test of the covariance. None of the two line-fits could reject the null hypothesis of a zero intercept, i.e. P = 0.6621 > α, whereas both line fits reject the null hypothesis with P = 0.0158 < α of a slope equal to that obtained when EP1 and EP2 data are grouped. Hence, a linear model of the type y = mx + 0 was used (Fig 2). The R^2^ values for both fits are 0.8259 and 0.9934 and the null hypothesis of a zero slope is rejected with P = 1.8218e-07 and 2.13e-04 < α for EP1 and EP2, respectively.

The covariance analysis leads to a significant difference between data from EP1 and EP2, with a P = 0.0158 < α, rejecting the null hypothesis of equal slopes. The slopes are 0.0424 and 0.1022 +/- 0.0113 standard error for the EP1 and EP2, respectively.

## Results

Fig 2 shows the number of viruses sampled with our ESP sampler, #Virus_ESP_, versus the number of viruses entering the sampler, #Virus_filter_. The corresponding raw measurement data can be found in Supplementary Information (S1 Table). Some of the measurements (typically when viral stock concentrations of less than 10^5^ RNA copies/mL were used) were below the LoD of the qPCR and were discarded. All blank control samples for cross-contamination control were below the LoD of the qPCR, verifying negligible cross-contamination between experiments.

The data show a linear relation between the number of viral RNA copies entering the sampler and those being collected in the sampler. When graphed in a log-log plot as in Fig 2, the linear fit model transforms into log y = log x + log m. The slope of the linear trend, *m*, reflects the collection efficiency. Data obtained with EP1 show a system collection efficiency of *η*_*coll*,*EP1*_ = 4.24%, while a significantly higher (P = 0.0158 < 0.05) collection efficiency *η*_*coll*,*EP2*_ = 10.21% is obtained with EP2. Only one measurement was successfully performed using EP3, giving an estimated collection efficiency of

$$\eta_{coll,EP3}=\;\;\frac{{\text{\#Virus}}_{\text{ESP}}}{{\text{\#Virus}}_{\text{filter}}}=47\%.$$

This clearly indicates that EP3 enables further improvement in the extraction efficiency. No significant difference was found between the different sample types (i.e., cultured H1N1, cultured H3N2, or clinical NP swabs H1N1).

The theoretical system LoD can be determined by considering the LoD of the qPCR assay, *LoD*_*qPCR*_ = 3 log RNA copies/mL, the liquid collector volume, *V*_*coll*_ = 150 μL, and the system collection efficiencies, *η*_*coll*,*EPi*_, as

$${\text{LoD}}_{EP_{i}}=\;\;\frac{{\text{LoD}}_{qPCR}\cdot V_{coll}}{\eta_{coll,EP_{i}}}.$$ (2)

The collection efficiency and the theoretical LoD for the different EPs are listed in Table 1 and are indicated in Fig 2.

**Table 1:** Summary of the data extrapolated from our measurements.

	System collection efficiency ($\eta_{coll,EP_{i}}$)	Relative improvement ($\eta_{coll,EP_{i}}/\eta_{coll,EP_{1}}$)	Theoretical Limit-of-Detection in # viral RNA copies in air (LoD_EPi_)
EP1	4.24% ± 1.13% s.e.m.	-	3341
EP2	10.21% ± 1.13% s.e.m.	2.4	1392
EP3	47%*	11*	303*
Theoretical max	100%	22	150

*data based on single measurement point.

In our experiments, the lowest amount of airborne virus that was tested and detected positive with our qPCR assay was 3721 RNA copies using EP1, a theoretical LoD of 1392 RNA copies using EP2, and 303 RNA copies using EP3. A maximum theoretical system LoD, i.e. at 100% collection efficiency, would be *LoD*_*max*_ = 150 viruses, as indicated with a dashed red arrow in Fig 2. All blank control measurements produced negative results, i.e. below the LoD of the qPCR, confirming that electrostatic precipitation is the effective sampling mechanism in our device and that it is a very effective means to capture virus-loaded aerosols.

The measurement error on the measurement data in Fig 2 was calculated to be 39% for filter measurements (x-axis) and 31% for ESP sampler measurements (y-axis). The source of these errors can be ascribed to the combined effect of the nebulization process, losses in the airflow tubing leading to the ESP sampler, and/or measurement errors. In the case of the measurements made with our ESP sampler, additional deviations may result from variations in aerosol charging in the ESP and variations in the EPs.

## Discussion

In this section, we first discuss the overall system performance, which derives from the simultaneous achievement of low overall system LoD and short sampling times. Next, we discuss our system performance and usefulness in view of practical PoC applications.

### Overall system LoD

As follows from Eqs 1 and 2, a low LoD requires the following: high collection efficiency *η*_*coll*_, i.e. high combined sampling efficiency, *η*_*samp*_, and extraction efficiency *η*_*extr*_; small sample dilution, i.e. small collection volume *V*; and low LoD in the viral detection assay (LoD_qPCR_).

### Collection efficiency

The results described in Fig 2 and Table 1 show that very satisfactory collection efficiencies can be obtained despite the miniaturized collection volume of 150 μL and the submicron aerosol size range. There is no observable statistical significant correlation between the system collection efficiency and viral subtype, viral load, or cultured strains versus clinical strains, demonstrating the robustness of the method in sampling airborne influenza virus.

As a comparison to our overall system collection efficiency, Agranovski et al. [3] reported a maximum system collection efficiency of 20% for influenza virus and 89% for *Vaccinia* virus using a “bubbler” sampler, although their work features limitations in terms of LoD capability due to the large collection volume (50 mL) and in terms of compatibility for integration in a PoC device.

### Sample dilution

With the miniaturized collection volume of 150 μL in our ESP sampler, the dilution of the sampled virus is reduced by at least one order of magnitude, as compared with other sampler types, such as impingers, which typically have a volume of 2 mL.

### Extraction efficiency

Experimental results indicate that both EP2 and EP3 yield improved viral collection by up to one order of magnitude when compared with EP1. This confirms our hypothesis that EP1 suffers from adsorption of viral particles to the inner and outer walls of the collector. This also indicates that the overall system collection efficiency is mostly limited by the extraction efficiency. Moreover, the physical sampling efficiency, even if it can be further improved by optimizing the device design, is relatively good. For this reason, future research should focus on optimizing the collector, specifically the choice of material and geometrical design, to minimize sample adsorption. Further research could also explore ways to automate and improve interfacing with a lab-on-chip to eliminate any handling and pipetting errors.

### LoD of our detection assay

As mentioned in the Experimental Approach in Section 2, the sampling-and-sensing scheme proposed here is inherently limited by the qPCR method and assay. Further improvements could come in the form of either improving this technique in terms of its LoD or replacing it with one achieving lower LoD, such as digital PCR. These changes would proportionally improve the capabilities of our system.

We believe that the overall system LoD may be further reduced by improvements in all the above described underlying parameters by improving collection efficiency, decreasing sample dilution, improving sample extraction, and providing more sensitive detection assays.

### Suggested minimum sampling time

For a given viral concentration in air, *c*_*sample*_, expressed as #viral particles/L, the minimum sampling time, *t*_*min*_, for positive detection directly derives from the overall system LoD, LoD_system_, the collection efficiency, *η*_*coll*_, and the sampling flow rate, *Q*_*samp*_, in L/min, as

$$t_{min}=\frac{{\text{LoD}}_{system}}{c_{sample}Q_{samp}\eta_{coll}}.$$

Hence, a high collection efficiency and low overall system LoD are critical factors in minimizing the overall sample-to-answer duration for diagnostics or monitoring applications, the other factors being rapid (integrated) sample handling and a rapid detection assay. Indeed, a rapid detection assay would be the critical factor in reducing sample-to-answer time from a few hours (dominated at present by the processing and analysis time of the qPCR method, namely, 1 to 8 hours) to a few minutes.

### Performance with relevance to practical applications

To give some perspective to the theoretical system LoD reported in Table 1 and to the sampling time needed for our approach, two possible real life applications are discussed: diagnostics of influenza using exhaled-breath and detection of influenza virus in indoor air for prevention of spread and epidemic monitoring.

For exhaled-breath diagnostics, Fabian et al. (2008) (in an observational study) reported detecting between 0 and 20 RNA copies/min in patients during normal tidal breathing using filters. Based on their data, our system should be able to detect influenza virus in exhaled breath by allowing patients to breathe for <16 minutes for the best estimated case of 20 RNA cp/min and EP3 in contrast to >3341 minutes ≈ 2 days 8 hours for the worse case of 1 RNA cp/min and EP1. The latter occurrence is clearly insufficient; however, the former looks very promising for exhaled breath diagnostics. It is promising especially when considering that exhaled-particle production can be increased by more than a magnitude factor, as compared to normal tidal breathing, when using special breathing maneuvers, as reported by Almstrand et al. [18].

For detection of influenza virus in indoor air, Yang et al. reported detecting influenza virus in eight of sixteen samples collected in both airplanes and day care centers using a cascade impactor with total virus concentrations ranging from 5800 to 37000 RNA copies per cubic meter of air [7]. Based on their data and using the sampling airflow rate of 6.7 L/min in our device, our technology should be able to detect influenza virus by sampling indoor air for < 2 minutes for the best estimated case of 37000 RNA cp/m^3^ and using EP3 and > 86 minutes for the worse case of 5800 RNA cp/m^3^ and using EP1, in which case, evaporation of the collection liquid would need special consideration. This is promising for future field studies enabling quantitative detection during epidemics.

It must be noted that the PCR-based detection employed in this work determines a quantitative value of virus particles based on genetic material, and since it does not probe the complete and intact virus particles, it is not a complete analysis of influenza virus in terms of infective particles [9]. In fact, standard infectivity assays typically quantify virus by determining the amount required to produce cytopathic effects in 50% of cultured cells. However, this method fails to account for the vast bulk of physical virions [24] and is an oversimplification of natural infection in humans. Generally, the ratio of intact virus particles to copies of nucleic acid is rarely one-to-one. In fact, no specific correlation has been shown between the two quantification methods for determining viral loads between qPCR and infectivity assays. Thus, we consider real time qPCR for our sampling-sensing scheme because it is not only much faster (typically 1–4 hours) than the TCID50 method (sometimes taking up to one week depending on cell infectivity time) but also it has a higher sensitivity than immunoassays, allowing detection of samples containing too few viruses to be detected with other methods.

Generally, there is a lack of data regarding viral loads in various settings, hospitals, emergency departments, common public spaces (airports, etc.), and enclosed spaces (cruise ships, etc.). These provide a great motivation to study the nature of airborne infectious diseases in such settings. For this application, our system may be suitable only if the bioanalysis is expanded by including infectivity or viability tests. Apart from this, our system may be applicable for use in several other applications. In addition to breath-based diagnostics, one may consider this solution for environmental monitoring of airborne infectious diseases (possibly Norovirus with the appropriate PCR assay, Rhinovirus, tuberculosis, etc.) for monitoring epidemics or diagnosing outbreaks. Additionally, such a scheme could be advantageous for biodefense and could even be used in support of a strategy to prevent bioterrorism or early identification of induced outbreaks.

Finally, with respect to the integration in a PoC instrument, our sampler design uses miniaturized liquid collector, which enables easy interfacing with a lab-on-chip detection cartridge. The high-voltage supplies and pumping systems can be made compact by packaging them into a shoebox size apparatus or smaller, using off-the-shelf components. A lab-on-chip cartridge reader may be needed to complement the system as a first step until further progress on miniaturization of the entire sensing and reading system is made, a development that will allow for a completely integrated system.

## Conclusion and outlook

In this work, we have demonstrated a technology suitable for the sampling and detection of sub-micron bioaerosols, specifically for detection of low concentration of airborne influenza viruses. We also discuss how our approach potentially enables detection of influenza virus in exhaled breath and in indoor air, with a sampling time of only a couple of minutes. Compared to other current technologies, our approach has the advantage to enable easy integration with lab-on-chip sensing technologies for point-of-care applications. Altogether, our results are promising towards “effective prevention and control strategies” during epidemics [4].

Future endeavors include optimizing the collector to minimize sample loss upon extraction and developing an ESP sampler with integrated lab-on-chip sensing technologies for use at point-of-care settings. Furthermore, field testing using the ESP sampler would contribute to improving our understanding of the natural state of airborne viruses, the viral load in various settings (hospital emergency rooms, clinics, prisons, breath samples, etc.), and specifics of the transmission pathway of influenza.

## Supporting information

S1 Fig Measurement of the particle size and mass distribution (left: absolute value per interval; left: relative value per interval) generated by the nebulizer under the operating conditions used for Aerosol A. (DOCX)

S2 Fig Data from Almstrand et al., 2010: Particle size and mass distribution (left: absolute value per interval; left: relative value per interval) measured in exhaled breath under various breathing patterns, as calculate from Table 3 in Almstrand et al., *Journal of Applied Physiology*, 2010. (DOCX)

S3 Fig Measurements of the amount of virus effectively reaching the inlet of our ESP, as measured using gelatin filters, versus the theoretical amount of virus that has been nebulized, based on the virus stock concentration and nebulized volume. (DOCX)

S4 Fig Plot of the mean value and confidence interval for the slopes of the linear regressions on the EP1 (y-axis label: 1) and EP2 (y-axis label: 2) data. The two slopes are statistically significantly different with a probability P = 0.0158 < α.
(DOCX)

S1 Table The amount of viral sample detected for different influenza subtypes, aerosolized concentrations, methods of aerosolization (indicated by data shaded in light grey), and methods of extraction (indicated by data shaded in dark grey). (DOCX)

S2 Table Improvement for Extraction Protocols 2 and 3 compared to Extraction protocol 1 for corresponding samples based on the results from Table 1. (DOCX)

S3 Table Linear regression parameters for the data groups obtained using EP1 and EP2. Covariance analysis for the data groups obtained using EP1 and EP2. Calculated using MATLAB covariance analysis function aoctool. The estimated intercept and slope for the data from EP1 and EP2, when considered individually, are indicated as deviations from the pooled-data values.
(DOCX)
