# Supplementary material for: Sampling and detection of airborne influenza virus towards point-of-care applications
Source: PLoS One. 2017 Mar 28;12(3):e0174314. doi: 10.1371/journal.pone.0174314 (PMC5369763; doi:10.1371/journal.pone.0174314)
Supplement: S1 Fig — (DOCX) [file pone.0174314.s001.docx]

***Aerosol generation***

**S1 Fig.** Measurement of the particle size and mass distribution (left: absolute value per interval; left: relative value per interval) generated by the nebulizer under the operating conditions used for Aerosol A.
