# Supplementary material for: Sampling and detection of airborne influenza virus towards point-of-care applications
Source: PLoS One. 2017 Mar 28;12(3):e0174314. doi: 10.1371/journal.pone.0174314 (PMC5369763; doi:10.1371/journal.pone.0174314)
Supplement: S2 Fig — (DOCX) [file pone.0174314.s002.docx]

**S2 Fig.** Data from Almstrand et al., 2010: Particle size and mass distribution (left: absolute value per interval; left: relative value per interval) measured in exhaled breath under various breathing patterns, as calculate from Table 3 in Almstrand et al., Journal of Applied Physiology, 2010.
