# Supplementary material for: Sampling and detection of airborne influenza virus towards point-of-care applications
Source: PLoS One. 2017 Mar 28;12(3):e0174314. doi: 10.1371/journal.pone.0174314 (PMC5369763; doi:10.1371/journal.pone.0174314)
Supplement: S3 Fig — (DOCX) [file pone.0174314.s003.docx]

**Upstream Losses**

The losses in the experimental setup upstream from the ESP sampler/Filters are plotted in S1 Fig, i.e. the measurement data of the filters, versus the amount of virus theoretically aerosolized from the stock sample (from the nebulized volume and qPCR measurement of the viral stock). A larger loss fraction is observable for low stock concentration, which we attribute to unspecific binding of the viruses to the surfaces in the nebulisation apparatus, i.e. in the syringe, in the tubing or in the nebulizer itself, effectively depleting low concentrated stock samples. Consequently, the nebulizer aerosolizes the supernatant of the stock sample, but not the virus themselves. Furthermore, the nebulizer was used in a single pass configuration, which means that larger particles are internally discarded with a built-in filter and are not recycled.

No specific difference can be attributed to the different virus types.


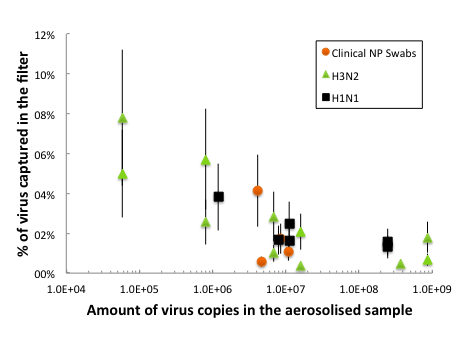


**S3 Fig.** Measurements of the amount of viruses effectively reaching the inlet of our ESP, as measured using gelatine filters, versus the theoretical amount of viruses that have been nebulized, based on the virus stock concentration and nebulized volume.
