# Supplementary material for: Sampling and detection of airborne influenza virus towards point-of-care applications
Source: PLoS One. 2017 Mar 28;12(3):e0174314. doi: 10.1371/journal.pone.0174314 (PMC5369763; doi:10.1371/journal.pone.0174314)
Supplement: S4 Fig — The two slopes are statistically significantly different with a probability P = 0.0158 < α. (DOCX) [file pone.0174314.s004.docx]

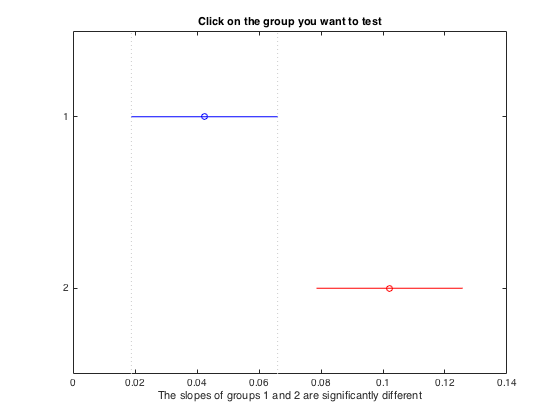


**S4 Fig.** Plot of the mean value and confidence interval for the slopes of the linear regressions on the EP1 (y-axis label: 1) and EP2 (y-axis label: 2) data. The two slopes are statistically significantly different with a probability P = 0,0158 < α.
