# Supplementary material for: Sampling and detection of airborne influenza virus towards point-of-care applications
Source: PLoS One. 2017 Mar 28;12(3):e0174314. doi: 10.1371/journal.pone.0174314 (PMC5369763; doi:10.1371/journal.pone.0174314)
Supplement: S1 Table — (DOCX) [file pone.0174314.s005.docx]

***Results***

S1 Table lists the results from all collected virus samples as the amount of viral RNA copies determined using qPCR. Samples for which the results are below the lower LoD of the qPCR are indicated as <LoD.

**S1 Table.** The amount of viral sample detected for different Influenza subtypes, aerosolized concentrations, methods of aerosolization (indicated by data shaded in light grey), and methods of extraction (indicated by data shaded in dark grey).

| **Sample name** | **Influenza Subtype** | **Aerosolized sample stock viral load (#copy/mL)** | **Nebulized volume and aerosol type** | **Collection Method** | **Extraction Protocol** | **#virus (#copy)**  **(duplicate measurements)** | |
| --- | --- | --- | --- | --- | --- | --- | --- |
| H1N1_AerA_Filt_1a;b | H1N1 | 6.03x10^4^ | 700 μL  Aerosol A | Filter | - | < LoD | < LoD |
| H1N1_AerA_EP1_1 |  |  |  | ESP | 1 | < LoD | < LoD |
| H1N1_AerA_Filt_2 |  | 1.70x10^6^ | 700 μL  Aerosol A | Filter | - | 4.54x10^4^ | < LoD |
| H1N1_AerA_EP1_2a;b |  |  |  | ESP | 1 | 1.90 x10^3^ | < LoD |
| H1N1_AerA_Filt_3a;b |  | 1.58x10^7^ | 700 μL  Aerosol A | Filter | - | 1.78 x10^5^ | 2.77 x10^5^ |
| H1N1_AerA_EP1_3a;b |  |  |  | ESP | 1 | 1.65 x10^3^ | 4.48 x10^3^ |
| H1N1_AerA_Filt_4 |  |  | 500 μL  Aerosol A | Filter | - | 1.32 x10^5^ | |
| H1N1_AerA_EP1_4 |  |  |  | ESP | 1 | 4.47 x10^3^ | |
| H1N1_AerA_EP2_4 |  |  |  | ESP | 2 | 1.05 x10^4^ | |
| H1N1_AerA_EP3_4 |  |  |  | ESP | 3 | 6.17 x10^4^ | |
| H1N1_AerA_Filt_5a;b |  | 3.55x10^8^ | 700 μL  Aerosol A | Filter | - | 3.87x10^6^ | 3.28 x10^6^ |
| H1N1_AerA_EP1_5a;b |  |  |  | ESP | 1 | 4.68 x10^4^ | 1.92 x10^4^ |
| H1N1_AerA_EP2_5 |  |  |  | ESP | 2 | 3.63 x10^5^ | |
| H3N2_AerA_Filt_1a;b | H3N2 | 8.32x10^3^ | 700 μL  Aerosol A | Filter | - | < LoD | < LoD |
| H3N2_AerA_EP1_1 |  |  |  | ESP | 1 | < LoD | < LoD |
| H3N2_AerA_Filt_2a;b |  | 8.32x10^4^ | 700 μL  Aerosol A | Filter | - | 4.54 x10^3^ | 2.90 x10^3^ |
| H3N2_AerA_EP1_2a;b |  |  |  | ESP | 1 | 2.97 x10^2^ | < LoD |
| H3N2_AerA_Filt_3a;b |  | 1.15x10^6^ | 700 μL  Aerosol A | Filter | - | 2.06 x10^4^ | 4.59 x10^4^ |
| H3N2_AerA_EP1_3a;b |  |  |  | ESP | 1 | 5.70 x10^2^ | 6.78 x10^2^ |
| H3N2_AerA_Filt_4a;b |  | 2.29x10^7^ | 700 μL  Aerosol A | Filter | - | 5.75 x10^4^ | 3.31 x10^5^ |
| H3N2_AerA_EP1_4a;b |  |  |  | ESP | 1 | 6.53 x10^3^ | 3.94 x10^3^ |
| H3N2_AerA_Filt_5 |  |  | 300 μL  Aerosol A | Filter | - | 6.92 x10^4^ | |
| H3N2_AerA_EP1_5 |  |  |  | ESP | 1 | 2.95 x10^3^ | |
| H3N2_AerA_EP2_5 |  |  |  | ESP | 2 | 1.35 x10^4^ | |
| H3N2_AerB_Filt_6 |  |  | 300 μL  Aerosol B | Filter | - | 1.95 x10^5^ | |
| H3N2_AerB_EP1_6 |  |  |  | ESP | 1 | 3.98 x10^3^ | |
| H3N2_AerA_Filt_7a;b |  | 1.23x10^9^ | 700 μL  Aerosol A | Filter | - | 1.54 x10^7^ | 5.74 x10^6^ |
| H3N2_AerA_EP1_7a;b |  |  |  | ESP | 1 | 3.20 x10^5^ | 7.25 x10^5^ |
| H3N2_AerA_Filt_8 |  |  | 300 μL  Aerosol A | Filter | - | 1.74 x10^6^ | |
| H3N2_AerA_EP1_8 |  |  |  | ESP | 1 | 2.00 x10^4^ | |
| H3N2_AerA_EP2_8 |  |  |  | ESP | 2 | 2.04 x10^5^ | |
| NP_AerA_Filt_1 | Clinical NP Swabs | 6.61x10^6^ | 700 μL  Aerosol A | Filter | - | 2.64 x10^4^ | |
| NP_AerA_EP1_1 |  |  |  | ESP | 1 | 1.73 x10^3^ | |
| NP_AerA_Filt_2 |  | 5.98 x10^6^ | 700 μL  Aerosol A | Filter | - | 1.70 x10^5^ | |
| NP_AerA_EP1_2 |  |  |  | ESP | 1 | 5.56 x10^3^ | |
| NP_AerA_Filt_3 |  | 1.23x10^7^ | 700 μL  Aerosol A | Filter | - | 1.49 x10^5^ | |
| NP_AerA_EP1_3 |  |  |  | ESP | 1 | 5.21 x10^3^ | |
| NP_AerA_Filt_4 |  | 1.55x10^7^ | 700 μL  Aerosol A | Filter | - | 1.18 x10^5^ | |
| NP_AerA_EP1_4 |  |  |  | ESP | 1 | 8.83 x10^3^ | |

The linear trends of Fig 3 were obtained by linear regression using the least-square method with forced zero-intercept of the data obtained using each EP. The assumption of a zero-intercept is supported by the fact that a non-zero-intercept has no real physical meaning, as follow: a positive y-axis intercept would mean that viruses are detected while no airborne virus is provided into the device; and a negative y-axis intercept would imply the existence of a lower threshold of influenza stock concentration, beyond which the sampler cannot efficiently collect the aerosol particles, while the particles themselves should remain unchanged.
