# Supplementary material for: Sampling and detection of airborne influenza virus towards point-of-care applications
Source: PLoS One. 2017 Mar 28;12(3):e0174314. doi: 10.1371/journal.pone.0174314 (PMC5369763; doi:10.1371/journal.pone.0174314)
Supplement: S2 Table — (DOCX) [file pone.0174314.s006.docx]

The potential improvements on a sample basis, summarized in S2 Table offered by the optimized EP 2 and 3 can be quantified basis as:

 (Eq 2)

**S2 Table.** Improvement for Extraction Protocols 2 and 3 compared to Extraction protocol 1 for corresponding samples, based on the results from Table 1.

| **Influenza Subtype** | **Aerosolized sample stock viral load (cp/mL)** | **Volume and size of aerosol droplets** | **Collection Method** | **Extraction Protocol** | **#virus (cp)** | **Improvement compared with Protocol 1** |
| --- | --- | --- | --- | --- | --- | --- |
| H1N1 | 1.58e7 | 500 μL  Aerosol A | Filter | - | 1.32 x10^5^ |  |
|  |  |  | ESP | 1 | 4.47 x10^3^ |  |
|  |  |  | ESP | 2 | 1.05 x10^4^ | 2.34 |
|  |  |  | ESP | 3 | 6.17 x10^4^ | 13.8 |
|  | 3.55e8 | 700 μL  Aerosol A | Filter | - | 3.87 x10^6^ |  |
|  |  |  | ESP | 1 | 4.68 x10^4^ |  |
|  |  |  | ESP | 2 | 3.63e5 | 18.9 |
| H3N2 | 8.32e3 | 300 μL  Aerosol A | Filter | - | 6.92e4 |  |
|  |  |  | ESP | 1 | 2.95e3 |  |
|  |  |  | ESP | 2 | 1.35e4 | 4.57 |
|  | 1.23e9 | 300 μL  Aerosol A | Filter | - | 1.74e6 |  |
|  |  |  | ESP | 1 | 2.00e4 |  |
|  |  |  | ESP | 2 | 2.04e5 | 10.2 |
