# Supplementary material for: Sampling and detection of airborne influenza virus towards point-of-care applications
Source: PLoS One. 2017 Mar 28;12(3):e0174314. doi: 10.1371/journal.pone.0174314 (PMC5369763; doi:10.1371/journal.pone.0174314)
Supplement: S3 Table — Covariance analysis for the data groups obtained using EP1 and EP2. Calculated using MATLAB covariance analysis function aoctool. The estimated intercept and slope for the data from EP1 and EP2, when considered individually, are indicated as deviations from the pooled-data values. (DOCX) [file pone.0174314.s007.docx]

***Data Analysis***

**S3 Table.** Linear regression parameters for the data groups obtained using EP1 and EP2. Covariance analysis for the data groups obtained using EP1 and EP2. Calculated using MATLAB covariance analysis function aoctool. The estimated intercept and slope for the data from EP1 and EP2, when considered individually, are indicated as deviations from the pooled-data values.

| **Regression coefficients** | | | | | |
| --- | --- | --- | --- | --- | --- |
| 'Term' | 'Estimate' | 'Std. Err.' | 'T' | 'Prob>\|T\|' |  |
| 'Pooled-data Intercept' | -3,44E+03 | 2,34E+04 | -0,1468 | 0,8848 |  |
| 'EP1' | -1,04E+04 | 2,34E+04 | -0,4439 | 0,6621 |  |
| 'EP2' | 1,04E+04 | 2,34E+04 | 0,4439 | 0,6621 |  |
| 'Pooled-data Slope' | 7,23E-02 | 1,13E-02 | 6,4138 | 3,77E-06 |  |
| 'EP1' | -2,99E-02 | 1,13E-02 | -2,6503 | 0,0158 |  |
| 'EP2' | 2,99E-02 | 1,13E-02 | 2,6503 | 0,0158 |  |
|  |  |  |  |  |  |
| **Analysis of covariance** | | | | | |
| 'Source' | 'd.f.' | 'Sum Sq' | 'Mean Sq' | 'F' | 'Prob>F' |
| 'ESP' | 1 | 3,56E+10 | 3,56E+10 | 8,8666 | 0,0077 |
| 'Filter' | 1 | 4,17E+11 | 4,17E+11 | 103,8101 | 3,89E-09 |
| 'ESP*Filter' | 1 | 2,82E+10 | 2,82E+10 | 7,0243 | 0,0158 |
| 'Error' | 19 | 7,63E+10 | 4,01E+09 | - | - |
